# A Comprehensive Transcriptome Analysis Identifies *FXN* and *BDNF* as Novel Targets of miRNAs in Friedreich’s Ataxia Patients

**DOI:** 10.1007/s12035-020-01899-1

**Published:** 2020-04-14

**Authors:** Julia O. Misiorek, Anna M. Schreiber, Martyna O. Urbanek-Trzeciak, Magdalena Jazurek-Ciesiołka, Lauren A. Hauser, David R. Lynch, Jill S. Napierala, Marek Napierala

**Affiliations:** 1 Institute of Bioorganic Chemistry, Polish Academy of Sciences, Poznan, Poland; 2 Department of Biochemistry and Molecular Genetics, UAB Stem Cell Institute, University of Alabama at Birmingham, Birmingham, AL USA; 3 Department of Pediatrics and Neurology, Perelman School of Medicine, University of Pennsylvania, Philadelphia, PA USA; 4 Children’s Hospital of Philadelphia, Philadelphia, PA USA

**Keywords:** Friedreich’s ataxia (FRDA), Frataxin (*FXN*), Brain-derived neurotrophic factor (*BDNF*), miRNAseq, RNAseq, microRNA-10a-5p, microRNA-224-5p

## Abstract

Friedreich’s ataxia (FRDA) is a genetic neurodegenerative disease that is caused by guanine-adenine-adenine (GAA) nucleotide repeat expansions in the first intron of the frataxin (*FXN*) gene. Although present in the intron, this mutation leads to a substantial decrease in protein expression. Currently, no effective treatment is available for FRDA, and, in addition to *FXN*, other targets with therapeutic potential are continuously sought. As miRNAs can regulate the expression of a broad spectrum of genes, are used as biomarkers, and can serve as therapeutic tools, we decided to identify and characterize differentially expressed miRNAs and their targets in FRDA cells compared to unaffected control (CTRL) cells. In this study, we performed an integrated miRNAseq and RNAseq analysis using the same cohort of primary FRDA and CTRL cells. The results of the transcriptome studies were supported by bioinformatic analyses and validated by qRT-PCR. miRNA interactions with target genes were assessed by luciferase assays, qRT-PCR, and immunoblotting. In silico analysis identified the *FXN* transcript as a target of five miRNAs upregulated in FRDA cells. Further studies confirmed that miRNA-224-5p indeed targets *FXN,* resulting in decreases in mRNA and protein levels. We also validated the ability of miRNA-10a-5p to bind and regulate the levels of brain-derived neurotrophic factor (*BDNF*), an important modulator of neuronal growth. We observed a significant decrease in the levels of miRNA-10a-5p and increase in the levels of *BDNF* upon correction of FRDA cells via zinc-finger nuclease (ZFN)-mediated excision of expanded GAA repeats. Our comprehensive transcriptome analyses identified miRNA-224-5p and miRNA-10a-5p as negative regulators of the *FXN* and *BDNF* expression, respectively. These results emphasize not only the importance of miRNAs in the pathogenesis of FRDA but also their potential as therapeutic targets for this disease.

**Electronic supplementary material:**

The online version of this article (10.1007/s12035-020-01899-1) contains supplementary material, which is available to authorized users.

## Background

Friedreich’s ataxia (FRDA, FA) is an autosomal recessive neurodegenerative disease that affects about 15,000 people worldwide, making it the most common inherited ataxia [1–4]. The vast majority of FRDA patients are homozygous for expanded GAA trinucleotide repeats in the first intron of the frataxin (*FXN*) gene. The expanded GAA repeats impede transcription of the *FXN* gene leading to severe downregulation of its mRNA and protein levels [5, 6]. Frataxin (FXN) is a small mitochondrial protein involved in the regulation of iron homeostasis and the biosynthesis of iron-sulfur clusters (Fe-S). Consequently, FXN deficiency disturbs intracellular iron metabolism [7] but, even more importantly, has broad cellular consequences via affecting the functions of numerous proteins requiring Fe-S clusters as prosthetic groups [8]. Therefore, processes such as mitochondrial respiration, energy metabolism, and DNA replication or repair are affected by decreased levels of FXN [9, 10]. FRDA affects many systems and organs, including the nervous system, both central and peripheral; heart; endocrine pancreas; vision; and hearing [11, 12]. The age of disease onset varies among patients and inversely correlates with GAA expansion length [13].

Given the crucial role of FXN in the biosynthesis of Fe-S clusters, numerous studies have reported significant transcriptome-wide changes in FRDA cells [14–16]. However, non-coding RNAs, including microRNAs (miRNAs), have not been extensively studied in FRDA cells despite the fact that research on miRNA biogenesis recurrently demonstrates a strong link between iron metabolism and miRNA synthesis and activity [17–21]. miRNAs are short (18–23 nt) non-coding RNAs, which bind predominantly to the 3′UTRs of complementary mRNAs and regulate their expression at the post-transcriptional level [22]. Extensive studies on neurodegenerative diseases have demonstrated important roles for miRNAs in the pathology of these diseases [23–25]. The first miRNA involved in FRDA pathogenesis was reported by Kelly et al. [26], who found, based on in silico studies, that miRNA-155 may be involved in a cardiac phenotype of FRDA. However, experimental studies did not confirm changes in this miRNA in the plasma of FRDA patients. Another study done using fibroblasts and lymphoblasts from FRDA patients indicated that miRNA-886-3p modulates *FXN* expression levels [27], which could potentially affect the severity of the FRDA phenotype. A follow-up study conducted in FRDA and control periodontal ligament cells demonstrated the opposite results, with miRNA-886-3p downregulated and miRNA-132 upregulated in FRDA cells [28], emphasizing the tissue/organ-related complexity of the miRNome. Subsequent studies on miRNA-886-3p reclassified this transcript as a significantly larger non-coding vault RNA transcribed by RNA polymerase III (vtRNA2-1, nc886) [29]. As miRNAs can be used as reliable disease biomarkers, researchers have also focused on defining FRDA-specific circulating miRNAs. Next-generation sequencing (NGS) and qRT-PCR validation identified seven miRNAs with elevated levels in FRDA plasma, among which miRNA-323-3p was proposed as a novel potential biomarker of cardiomyopathy progression. *ATP2A2* (ATPase sarcoplasmic/endoplasmic reticulum Ca^2+^ transporting 2) was identified as a target of miRNA-323-3p, which could be involved in and have an impact on cardiomyopathy progression [30]. A set of miRNAs differentially expressed in FRDA plasma was also defined by Dantham et al., who applied microarray and qRT-PCR analyses in a miRNA screen [31]. No commonly changed miRNAs were identified between these two last studies, which may be due to different ethnic origin (Spanish versus Indian) or methodology used for miRNA profiling. In addition, the work of Seco-Cervera et al. specifically focused on changes in miRNA expression in association with cardiomyopathy [30], the most frequent cause of death in FRDA.

In the present study, we performed NGS of miRNAs in 15 FRDA and 15 control (CTRL) primary fibroblast cell lines for which we had previously generated comprehensive transcriptome data [14] and conducted integrated transcriptome analysis of both datasets. We validated the differentially expressed miRNAs and did bioinformatic analyses of their potential targets. We identified *FXN* and *BDNF* as targets of miRNAs whose expression levels were elevated in FRDA cells compared to the CTRL cells. These results demonstrate the therapeutic potential of targeting miRNAs to either increase *FXN* expression or indirectly alleviate the consequences of reduced levels of *FXN*.

## Methods

### Cell Culture and Transfections

Primary fibroblast isolation was performed as described previously [14]. Characterization data of the FRDA and CTRL fibroblast lines used in the studies is presented in Table 1. All studies using patient and control cell lines were approved by the Children’s Hospital of Philadelphia (CHOP) and University of Alabama (UAB) Institutional Review Boards (CHOP IRB #10-007864; UAB IRB #N131204003). Fibroblasts, HEK293, and HeLa cells were grown in DMEM high glucose medium (Gibco, Thermo Fisher Scientific, Carlsbad, CA) supplemented with 15% fetal bovine serum (Biowest, Riverside, MO), l-glutamine (Gibco, Thermo Fisher Scientific, Carlsbad, CA), non-essential amino acids (Life Technologies, Carlsbad, CA), and antibiotic-antimycotic (Sigma-Aldrich, St. Louis, MO). No differences in population doubling time or increased cellular death or senescence between FRDA and CTRL primary fibroblasts were observed under our standard culture conditions. Transfection was performed using Lipofectamine2000 (Thermo Fisher Scientific, Carlsbad, CA) for HEK293 and HeLa cells or the Neon system (Thermo Fisher Scientific, Carlsbad, CA) for HeLa cells according to the manufacturer’s recommendations. Transfection efficiency was monitored by parallel transfections with pmaxGFP (Lonza, Rockland, ME; for plasmid transfection) and BLOCK-iT™ Fluorescent Oligo (Thermo Fisher Scientific, Carlsbad, CA; for miRNA mimics or inhibitors).

**Table 1:** Characterization of the FRDA and CTRL fibroblast lines used for miRNA sequencing

Cell Line	Sex	Sampling Age (years)	*FXN* level vs AVG CTRL (RNA-seq)	Age of disease onset (years)	No. of GAA repeats (allele 1, allele 2)	CMP	Diabetes	FARS score (age at exam, years)
FRDA								
68	F	21	0.3	7	570, 1200	+	–	98 (24)
88	F	50	0.42	16	422, 520	–	–	75 (52)
203	F	31	0.34	14	916, 1382	–	–	88 (32)
4230	F	28	0.22	6	870, 1470	+	+	101 (31)
4497	F	44	0.38	30	526, 826	–	–	59.5 (46)
4627	F	50	0.44	22	501, 670	–	–	84.5 (51)
281	M	19	0.3	11	630, 806	+	–	75.5 (20)
4192	M	33	0.32	16	400, 967	–	–	63 (36)
156	M	41	0.34	15	495, 505	–	–	85.5 (43)
188	M	47	0.45	11	490, 680	–	–	95 (47)
4259	M	37	0.46	15	404, 920	+	–	ND
4509	M	36	0.47	18	211, 1428	–	–	38 (37)
4654	M	19	0.51	16	190, 500	+	–	30.3 (21)
4675	M	28	0.38	4	185, 1130	–	–	69.5 (28)
4743	M	29	ND	16	498, 1043	+	–	54 (31)
Female	6							
Male	9							
Mean		34	0.4*	14.5	492, 937			73 (36)
Median		33	0.38	15	497, 920			75 (34)
CTRL								
7522^1^	F	19	1.09	N/A	N/A	N/A	N/A	N/A
5879^1^	F	48	1.07	N/A	N/A	N/A	N/A	N/A
3956^1^	F	27	1.11	N/A	N/A	N/A	N/A	N/A
2036^1^	F	11	0.91	N/A	N/A	N/A	N/A	N/A
1650^1^	F	37	0.88	N/A	N/A	N/A	N/A	N/A
7525^1^	F	22	0.99	N/A	N/A	N/A	N/A	N/A
8399^1^	F	19	0.93	N/A	N/A	N/A	N/A	N/A
C_UF	F	35	1.09	N/A	N/A	N/A	N/A	N/A
7492^1^	M	17	0.9	N/A	N/A	N/A	N/A	N/A
3348^1^	M	10	0.89	N/A	N/A	N/A	N/A	N/A
2153^1^	M	40	1.23	N/A	N/A	N/A	N/A	N/A
288^1^	M	64	1.02	N/A	N/A	N/A	N/A	N/A
3652^1^	M	24	0.92	N/A	N/A	N/A	N/A	N/A
C_UM	M	39	1.32	N/A	N/A	N/A	N/A	N/A
21808^1^	M	1	1.19	N/A	N/A	N/A	N/A	N/A
Female	8							
Male	7							
Mean		28	1					
Median		24	1.02					

No significant difference exists between the FRDA and CTRL groups for sampling age as determined by Student’s *t* test

*N/A* not analyzed, *ND* not determined, *CMP* cardiomyopathy, *+* present, *−* absent, *FARS* Friedreich’s Ataxia Rating Scale

*Significant difference (*p* < 0.001) in *FXN* mRNA levels between CTRL and FRDA groups

^1^FRDA and CTRL fibroblast lines obtained from Coriell Repositories

### RNAseq and miRNAseq

Cells were harvested at 70–90% confluence. Total RNA and total miRNA were isolated by a Qiagen RNeasy Mini Kit and miRNeasy Mini Kit, respectively (Qiagen, Hilden, Germany) according to the manufacturer’s recommendation. RNAseq was performed as described previously [14]. MicroRNA profiling was conducted using the Illumina HiSeq 2500 system. Briefly, total RNA quality was assessed on the Agilent BioAnalyzer, and the presence of a strong 5S peak indicated retention of the miRNA fraction. Next-generation sequencing libraries were produced using the TruSeq small RNA library prep kit (Illumina, San Diego, USA). The miRNA fraction was used as a substrate for adaptor ligation at the 3′ end through an RNA ligase directed mechanism. The 3′ ligation was followed by the addition of a 5′ adaptor and reverse transcription to generate first-strand cDNA. An initial PCR step was performed to produce the 2nd strand and to introduce unique indexes to each sample, sequences necessary for flow cell attachment and sequencing. Finally, the libraries were purified with magnetic beads and quantitated using the Kapa Biosystems qPCR quantitation for Illumina libraries. The resulting libraries were standardized for concentration and sequenced on the HiSeq 2500 system using 50 bp single end reads at UAB Heflin Center for Genomic Sciences. RNAseq data files are available at GEO accession GSE104288 [32]. Sequencing reads (> 15,000,000 per sample) after trimming were mapped using TopHat (v2.0.13). The DESeq (v3.0) package was used for differential expression analysis. DESeq counts were used to calculate log2 expression values for miRNA genes and their mRNA targets. A Python matplotlib library was used to generate heatmaps, scatter plots, and correlation plots. For generation of heatmaps, data were normalized by subtracting the median and division by the maximum value minus the median that resulted in values in the range (− 1, 1) per gene. Regression lines between normalized expression levels of miRNAs and target genes were calculated with least-squares regression for two sets of measurements (using the linregress function from scipy.stats library). Correlations were performed for the reduced group of samples: C1650, C2153, C21808, C288, C3348, C3652, C3956, C5879, C7492, C7522, C7525, C8399, F156, F188, F203, F281, F4192, F4230, F4259, F4497, F4509, F4627, F4675, F68, and F88. The significance of the correlation between expression levels was calculated with the Spearman rank-order correlation test (using Spearman’s function from the scipy.stats library). For statistical analysis, *P* ≤ 0.05 was considered significant. Calculations of Venn diagram data were performed with a set of in-house Python scripts.

### RNA Extraction, qRT-PCR with SYBR Green, and TaqMan Probes

Cells were harvested at 70–90% confluence. Total RNA and miRNA were isolated by Qiagen RNeasy Mini Kit (Qiagen, Hilden, Germany) or miRNeasy Mini Kit (Qiagen, Hilden, Germany) followed by rigorous DNase I treatment. RNA (1–2 μg) was reverse transcribed into cDNA using a High Capacity cDNA Reverse Transcription Kit with RNase inhibitor (Applied Biosystems, Foster City, CA) and a cDNA dilution (1:2–1:100). Both TaqMan (Applied Biosystems, Thermo Fisher Scientific, Carlsbad, CA) and SYBR Green (Bio-Rad, Hercules, CA) approaches were used to verify gene expression. The cDNA synthesis reactions for miRNA analyses were performed using 10 ng of total RNA with a TaqMan Advance miRNA cDNA Synthesis Kit (Applied Biosystems, Thermo Fisher Scientific, Carlsbad, CA). miRNA expression was verified by qRT-PCR with specific TaqMan probes according to the manufacturer’s protocol using a Bio-Rad CFX96 thermocycler (Bio-Rad, Hercules, CA). All primers and TaqMan probes used are listed in Table 2.

**Table 2:** TaqMan gene expression probes purchased from Thermo Fisher Scientific and primer sequences used in SYBR Green analyses

miRNA/target gene	Assay ID/primer sequence
miR-10a-5p	479241_mir
miR-26a-5p	477995_mir
miR-148a-3p	477814_mir
miR-193a-3p	478306_mir
miR-212-5p	478767_mir
miR-224-5p	483106_mir
miR-3607-5p	478835_mir
miR-7641	479172_mir
*BDNF*	Hs02718934_s1
*GAPDH*	Hs03929097_g1
*FXN* forward	5′CAGAGGAAACGCTGGACTCT3′
*FXN* reverse	5′AGCCAGATTTGCTTGTTTGG3′
*GAPDH* forward	5′GAAGGTGAAGGTCGGAGTC3′
*GAPDH* reverse	5′GAAGATGGTGATGGGATTTC3′

### Immunoblotting

Samples for the protein analyses were harvested and extracted in a buffer containing 0.1% NP-40, 0.25 M NaCl, 5 mM EDTA, 50 mM HEPES of 7.5 pH, 0.1%, 0.5 mM DTT, and protease inhibitor cocktail (Sigma-Aldrich, St. Louis, MO). Protein concentrations were assessed by spectrophotometry at 280 nm (DeNovix, Wilmington, DE), and 40 μg of protein extract was loaded onto an SDS-PAGE gel, electrophoresed, transferred to a 0.1-μm nitrocellulose membrane (GE Healthcare, Chicago, IL), and immunoblotted with anti-FXN mouse antibody 17A11 (Abcam, Cambridge, UK) or anti-GAPDH mouse 6C5 antibody (Merck, Darmstadt, Germany) followed by immunoblotting with peroxidase conjugated donkey anti-mouse IgG antibody (Jackson ImmunoResearch Laboratories, West Grove, PA). Quantitation of immunoblots was performed using ImageJ software (National Institutes of Health, Bethesda, MD).

### Luciferase Assays

A luciferase assay was performed for two variants depending on the cell line used. HEK293 and HeLa cells were transfected with miRNAs at a final concentration of 15 nM and 100 nM, respectively, together with 50 ng constructs containing the predicted miRNA target sequence or its mutated variant cloned into the *PmeI* and *XbaI* restriction sites of the pmirGLO vector (Promega, Madison, WI). Forty-eight hours after transfection, the cells were lysed in passive lysis buffer (Promega, Madison, WI), and the luciferase activity was measured using a Dual-Glo Luciferase Assay System (Promega, Madison, WI) on a VictorX4 Multilabel plate reader (PerkinElmer, Waltham, MA). *Firefly* luciferase activity was normalized to *Renilla* luciferase activity for each construct and compared to the pmirGLO vector without the insert. All calculations were averaged from at least three independent experiments. The sequences of miRNA binding sites cloned into luciferase reporter constructs are listed in Suppl. Tab. 1.

### GAA Editing by ZFNs

Editing was performed as described previously [33]. Briefly, FRDA fibroblasts hemizygous for GAA expansion (one allele with expanded GAAs and a second edited by Zinc-finger nucleases (ZFNs)) were transfected with RNA encoding ZFN-UP and ZFN-DN. A homozygous edited clone with both GAA tracts excised was identified by PCR [33] and used in the present studies. Increased expression of *FXN* mRNA upon homozygous GAA excision was confirmed using qRT-PCR.

### Statistical Analysis

Statistical calculations, with the exception of the RNAseq and miRNAseq analyses, were performed by using Excel and GraphPad PRISM with Student’s *t* test. Two-tailed *P* values below or equal 0.05 were considered significant: *P ≤ 0.05, ***P ≤ 0.001.

## Results

### miRNA Signature of FRDA Cells

**Fig. 1:**
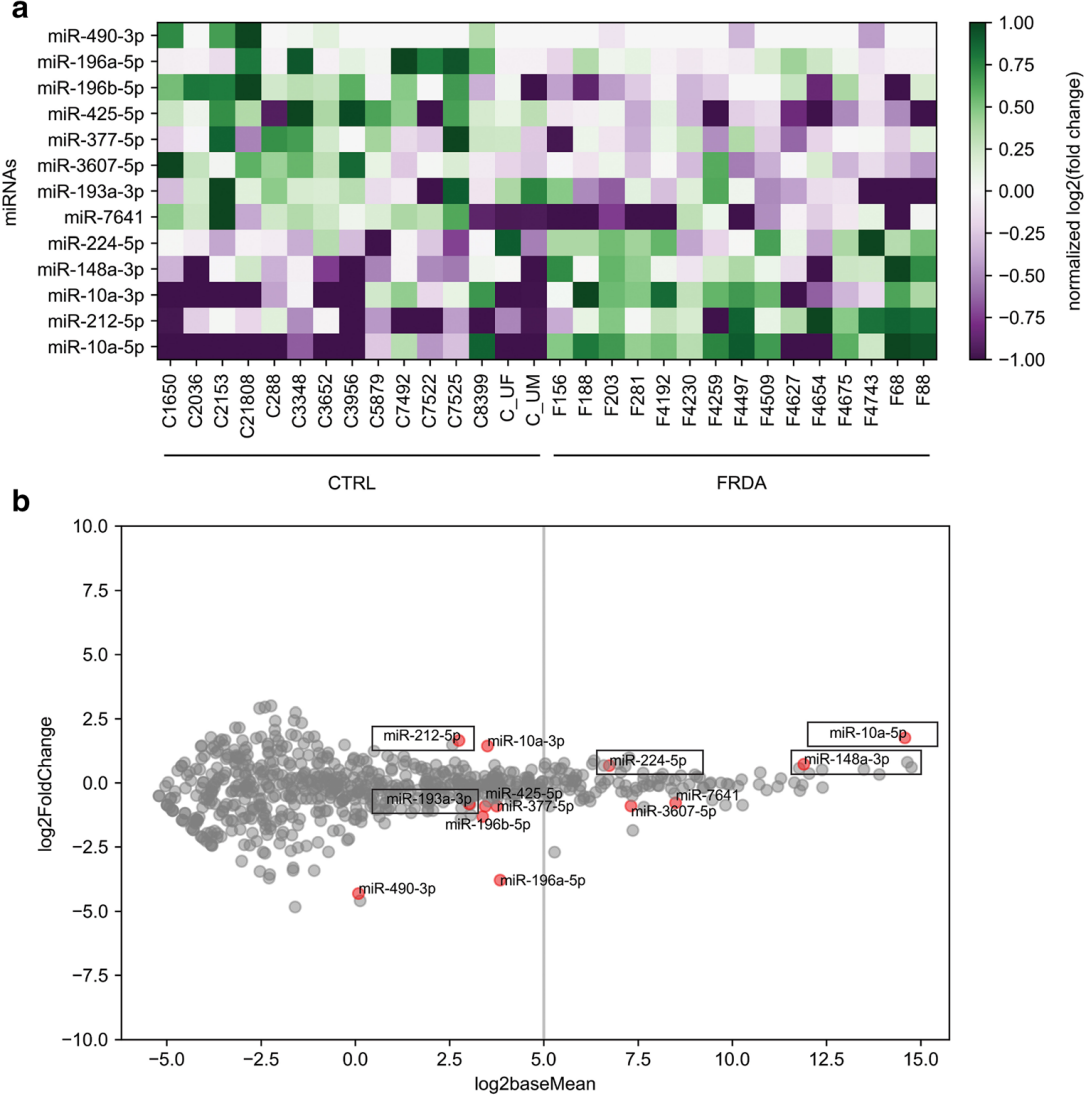
Quantitative miRNA profiling. **a** A heatmap was generated based on the normalized DESeq counts and shows the miRNA expression levels from CTRL and FRDA patient fibroblasts (each group *n* = 15). miRNAs were selected based on statistically significant (*P ≤ 0.05) log2-fold changes between the groups. Expression levels are indicated by colored bars from purple (low expression) to green (high expression). **b** Scatter plot illustrating the fold change in miRNA expression between the CTRL and FRDA patient *groups* as log2-transformed data. Differentially expressed miRNAs in FRDA fibroblasts are shown as red dots. The gray vertical line indicates a cutoff of separating the miRNAs with higher expression levels (log2baseMean > 5.0) from those with lower expression levels (log2baseMean < 5.0). Based on this distinction, a pool of highly expressed miRNAs was selected for further analysis. Boxes designate miRNAs that, according to the TargetScan 7.2 database, are predicted to target the *FXN* gene

To characterize miRNA expression in FRDA cells, we utilized unbiased RNA sequencing of the small RNA fraction (miRNAseq). The analysis was performed on primary fibroblasts from both FRDA and non-disease carriers, CTRL, each group consisting of 15 cell lines, which were deposited in our laboratory [34]. Characterization data of the fibroblast lines, including sex, age at sampling and age of onset, the GAA repeat number, *FXN* level, as well as the development of cardiomyopathy, diabetes, and FARS scores, is presented in Table 1. Differentially expressed miRNAs (statistically significant difference *P ≤ 0.05, base mean) between the FRDA and CTRL groups (DEseq) were identified from the pool of all 1059 sequenced miRNAs (Fig. 1a). miRNAseq identified a total of 13 differentially expressed miRNAs: 5 upregulated and 8 downregulated in the FRDA group compared to the CTRL group (Fig. 1a, b).

For further validation and analyses, we selected 5 miRNAs: miRNA-10a-5p, miRNA-148a-3p, miRNA-7641, miRNA-3607-5p, and miRNA-224-5p that demonstrated the highest expression levels (normalized RNAseq signal > 100) (Fig. 1b). miRNA-10a-5p, miRNA-148a-3p, and miRNA-224-5p were upregulated in the FRDA cohort while miRNA-7641 and miRNA-3607-5p were downregulated in FRDA samples. We included two additional differentially expressed miRNAs: miRNA-193a-3p and miRNA-212-5p. These two miRNAs, although expressed at low levels, were predicted in silico to target the *FXN* mRNA.

**Fig. 2:**
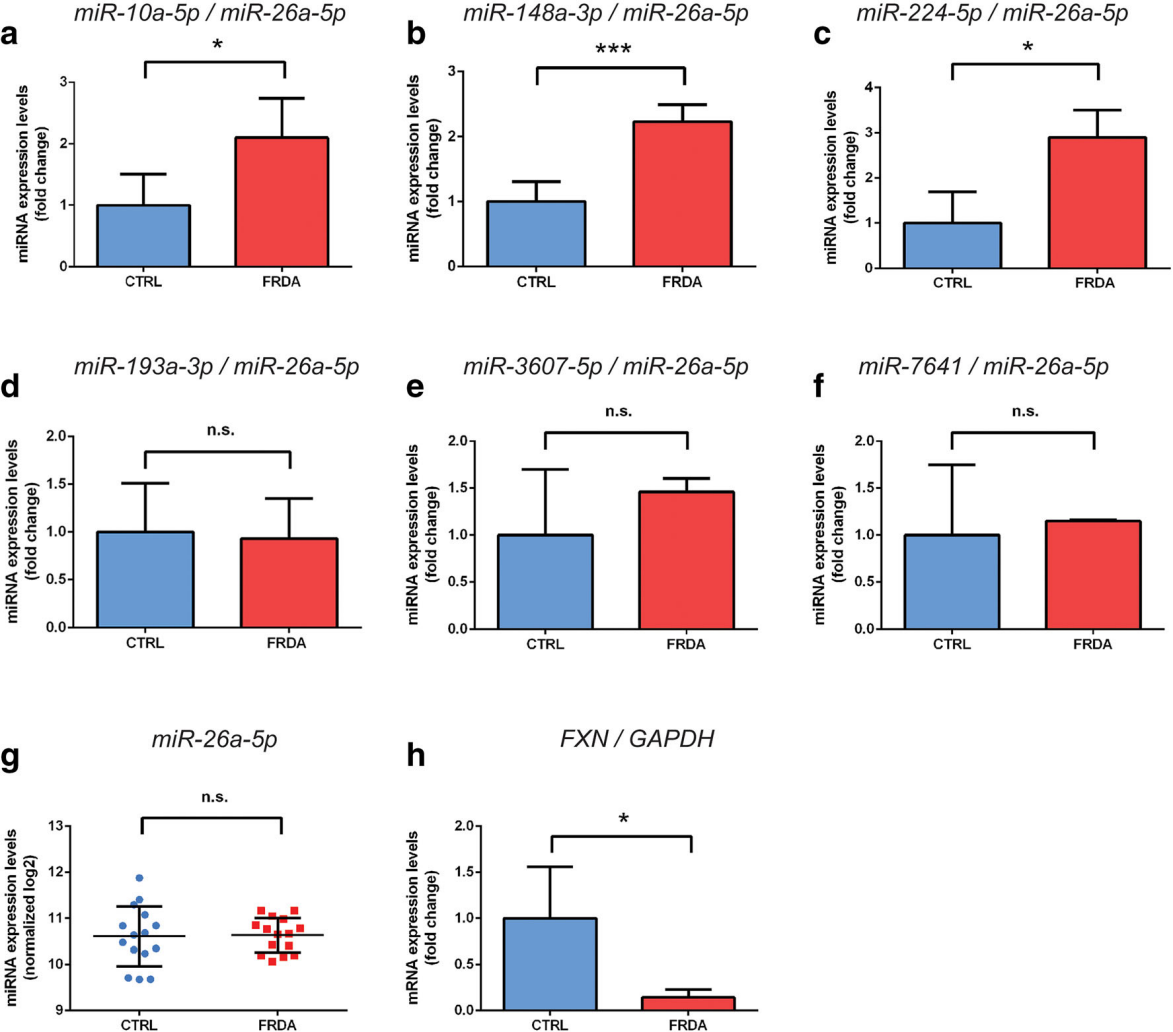
Validation of the miRNA sequencing results and determination of *FXN* levels by qRT-PCR. **a**–**c** The expression levels of miRNA-10a-5p, miRNA-148a-3p, and miRNA-224-5p were upregulated in the FRDA compared to CTRL fibroblasts. **d**–**f** The expression levels of miRNA-193a-3p, miRNA-3607-5p, and miRNA-7641 were not changed in the FRDA compared to CTRL fibroblasts. miRNA-212-5p was not detected by qRT-PCR (not shown). **g** miRNA expression levels were normalized to miRNA-26a-5p, which is uniformly expressed in both cohorts. Log2 values for each sample are presented as blue (CTRL) and red (FRDA) dots. Horizontal lines indicate a mean value for each group. **h** Decreased expression level of *FXN* in FRDA fibroblasts. Comparisons were performed using unpaired Student’s *t* tests. *P ≤ 0.05, ***P ≤ 0.001, n.s. non-significant. Bars present the normalized average fold change compared to the CTRL fibroblasts (5 cell lines/group) with the standard deviation (SD)

qRT-PCR analyses performed on 5 FRDA and 5 CTRL fibroblast lines confirmed the significant differences in expression between the FRDA and CTRL groups for miRNA-10a-5p, miRNA-148a-3p, and miRNA-224-5p (Fig. 2a–c). Notably, miRNA-10a-5p and miRNA-148a-3p displayed the highest overall expression levels among all miRNAs detected in miRNAseq (Fig. 1b). We were unable to validate the differential expression of miRNA-193a-3p, miRNA-3607-5p, and miRNA-7641 with the qRT-PCR technique, while the signal from miRNA-212-5p was not detectable by qRT-PCR (Fig. 2d–f). miRNA-26a-5p was used as an endogenous control in all miRNA qRT-PCR analyses due to its comparable expression levels between the CTRL and FRDA groups in the miRNAseq experiment (Fig. 2g). The *FXN* mRNA expression levels in fibroblast lines were confirmed using qRT-PCR (Fig. 2h).

### *FXN* as a Potential Target of miRNAs Differentially Expressed in FRDA Fibroblasts

**Fig. 3:**
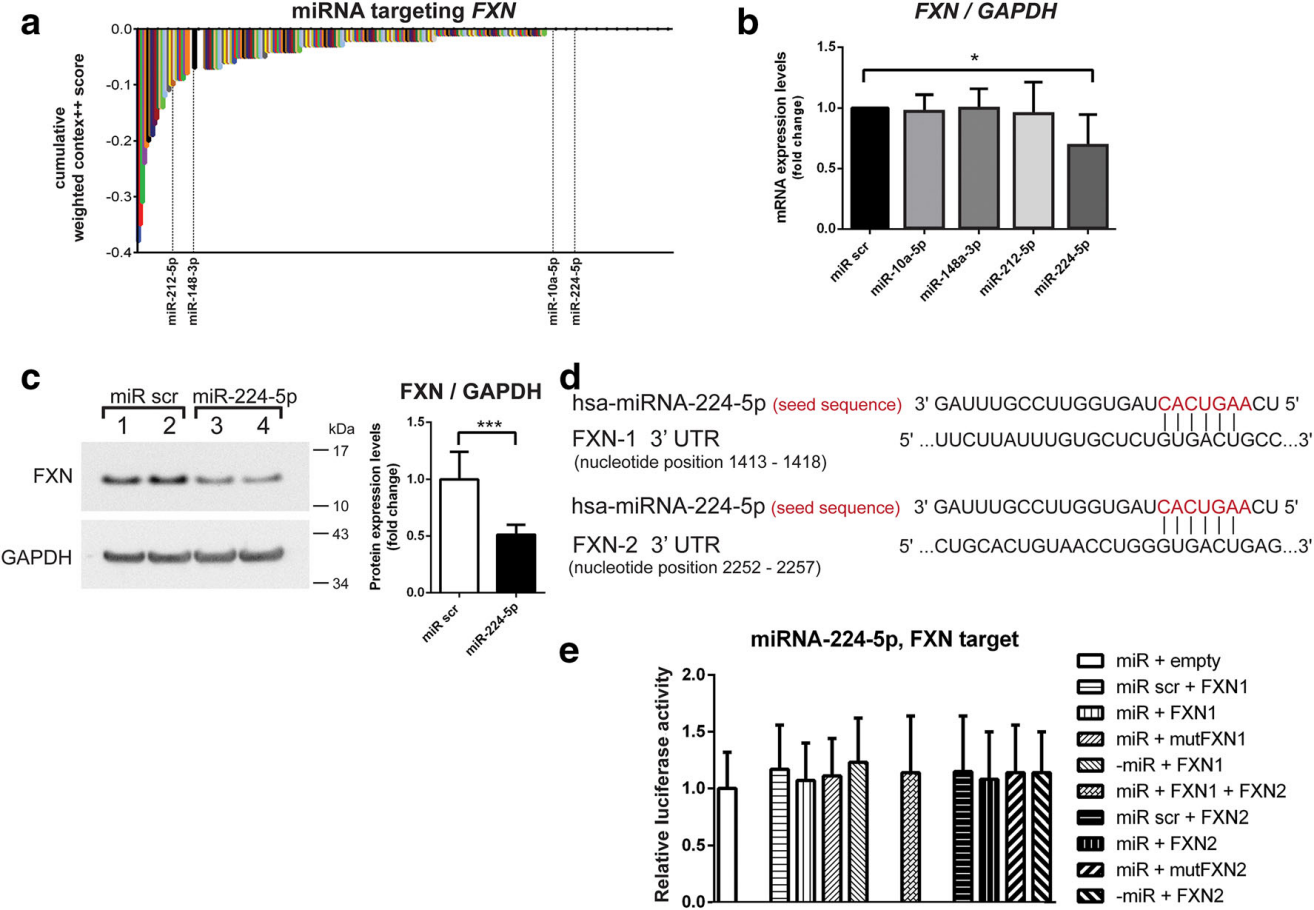
Regulation of *FXN* expression by miRNA-10a-5p, miRNA-148a-3p, miRNA-212-5p, and miRNA-224-5p. **a** The waterfall chart presents the miRNAs targeting the *FXN* gene positioned by the increased cumulative weighted context++ from the TargetScan 7.2 database. The differentially expressed miRNAs in FRDA fibroblasts are indicated on the graph with dashed lines. **b** Expression of *FXN* mRNA in HeLa cells transfected with *FXN* targeting miRNAs: miRNA-10a-5p, miRNA-148a-3p, miRNA-212-5p, and miRNA-224-5p, as determined by qRT-PCR. miR scr represents the *C. elegans* miRNA cel-miR-239b used as a negative control. **c** Analysis of FXN protein level using immunoblotting after transfection of HeLa cells with miRNA-224-5p. Quantitation of immunoblotting results is presented in the right panel. Data were collected from three independent experiments. **d** Visualization of human miRNA-224-5p binding to two sites of the *FXN* 3′UTR. The seed sequence of the miRNA is indicated in red, and the exact locations of the putative target sequences in the *FXN* 3′UTR are indicated (based on NM_000144.4, nucleotide positions: *FXN1* 1413–1418 and *FXN2* 2252–2257). **e** Two fragments of the *FXN* 3′UTR (FXN1 and FXN2) harboring putative target sequences for miRNA-224-5p were cloned into separate reporter vectors. Constructs with mutated seed sequences of the targets and non-targeting miRNA were used as negative controls. HeLa cells were co-transfected with generated constructs (50 ng) and synthetic miRNAs (100 nM). Luciferase activity was measured 48 h after transfection. *Firefly* luciferase activity was normalized against *Renilla* luciferase activity. All bars present the relative luciferase activity with the standard deviation (SD). Normalization was performed for miRNA-224-5p co-transfected with a plasmid lacking a target sequence (“miR + empty”). Statistical significance was determined using Student’s *t* test *P ≤ 0.05, ***P ≤ 0.001

**Fig. 4:**
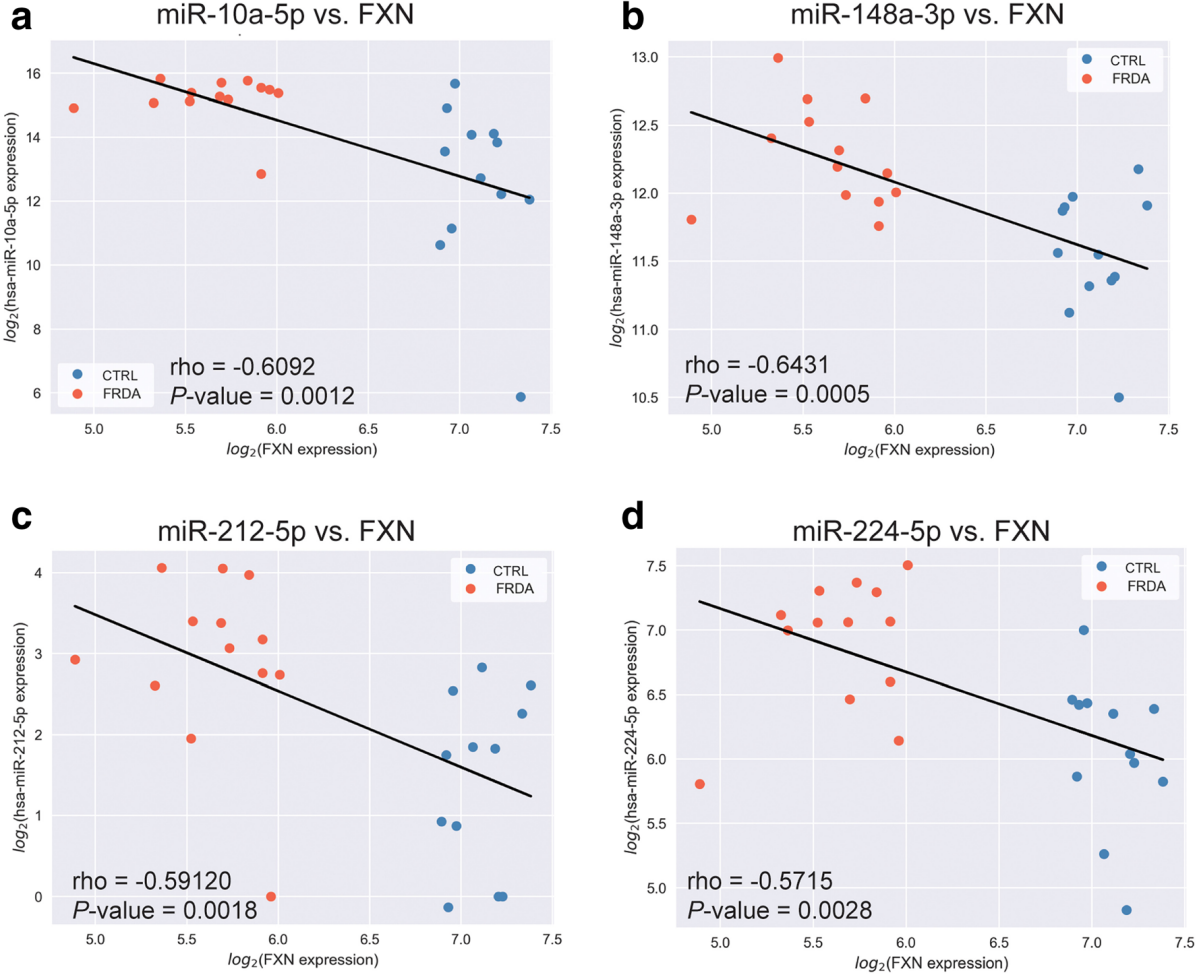
Correlation between the expression of miRNAs targeting the *FXN* gene, and *FXN* expression levels. **a**–**d** A negative Spearman’s correlation was calculated for the miRNA-10a-5p, miRNA-148a-3p, miRNA-212-5p, miRNA-224-5p, and *FXN* expression levels. The regression line between the normalized expression of the miRNA and the *FXN* gene was calculated with least-squares regression for two sets of measurements. CTRL samples from non-disease carriers are shown as blue dots, and FRDA patient samples are shown as red dots. Spearman’s rho and *P* values are indicated

We identified a set of five miRNAs: miRNA-10a-5p, miRNA-148a-3p, miRNA-193a-3p, miRNA-212-5p, and miRNA-224-5p; these miRNAs showed altered expression levels in FRDA cells (by miRNAseq) and that were predicted by TargetScan 7.2 [35] to target *FXN* mRNA (Figs. 1b and 3a). A strong negative correlation between the expression levels of these miRNAs, except for miRNA-193a-3p (which did not pass the qRT-PCR validation), and *FXN* mRNA was observed (Fig. 4a–d). Based on the TargetScan cumulative weighted context++ score (analysis of the total repression of predicted mRNA targets by multiple sites of the same miRNA), miRNA-10a-5p, miRNA-148a-3p, miRNA-212-5p, and miRNA-224-5p exhibited different likelihoods of targeting *FXN* mRNA (Fig. 3a). Next, we experimentally tested whether these miRNAs can affect the expression of *FXN*. We transfected HeLa cells, which, according to the human miRNA expression database (miRmine, [36]), express the studied miRNAs at relatively low levels, with synthetic miRNAs corresponding to these miRNAs and evaluated *FXN* mRNA levels by qRT-PCR (Fig. 3b). A statistically significant decrease in *FXN* mRNA and FXN protein levels was observed only upon has-miRNA-224-5p treatment, as shown by qRT-PCR and immunoblot analyses, compared to that following transfection with the control cel-miR-239b (miR scr, a *Caenorhabditis elegans* miRNA) (Fig. 3b, c). To determine whether miRNA-224-5p can directly target *FXN*, we performed a luciferase reporter assay in HeLa cells. In silico analysis of the *FXN* 3′UTR site revealed two putative binding sites for miRNA-224-5p; thus, two luciferase vectors were designed, each with a separate 3′UTR binding site (Fig. 3d). Firefly luciferase vectors, empty or containing the wild-type *FXN* 3′UTR or the mutant *FXN* 3′UTR (mutations in putative miRNA binding sites), were co-transfected with synthetic hsa-miR-224-5p or cel-miR-239b, followed by luciferase expression assays [37]. The sequences of the miRNA binding sites cloned into luciferase assay constructs are presented in Suppl. Tab. 1. The analyses revealed no effect of synthetic hsa-miRNA-224-5p on the expression of the luciferase constructs with an inserted fragment of the *FXN* 3′UTR when compared to that of the empty vector control (Fig. 3e). Thus, the discrepancy between the results of the hsa-miRNA-224-5p transfection on the endogenous *FXN* levels and the luciferase constructs containing putative miR-224-5p binding sites indicate a potential indirect effect of this miRNA on *FXN* expression.

### Integrative Analysis of miRNome and Transcriptome Profiling Data

**Fig. 5:**
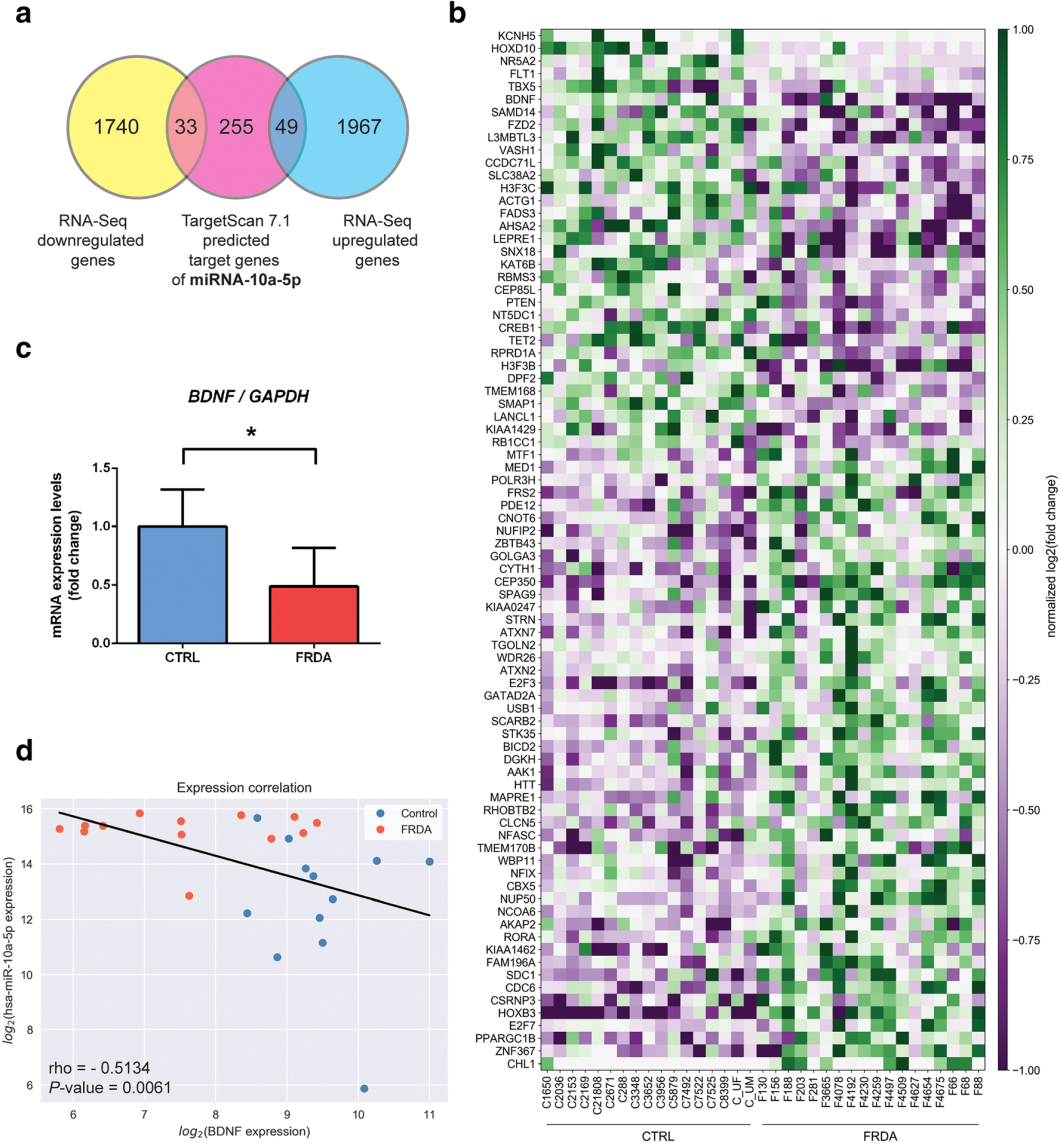
miRNA-10a-5p overexpression in FRDA targets *BDNF*. **a** Identification of miRNA-10a-5p targets. Venn diagram illustrates an overlap between mRNAs that were differentially expressed in FRDA fibroblasts (yellow—downregulated, blue—upregulated) and TargetScan 7.1 predicted targets of miRNA-10a-5p (red). **b** A heatmap presents the expression of 82 genes (33 downregulated and 49 upregulated, *P ≤ 0.05 in unpaired Student’s *t* test) predicted to be targets of miRNA-10a-5p in FRDA (*n* = 18) and CTRL (*n* = 17) fibroblasts. The expression level is represented by the colored bars from purple (low expression) to green (high expression). **c***BDNF* expression is decreased in FRDA cells. Validation of RNAseq data by qRT-PCR. Bars represent the fold change between the FRDA and CTRL fibroblasts (5 cell lines/group); the results were normalized to *GAPDH* expression; error bars represent the standard deviation of the mean (SD). **d** Negative correlation between the expression of miRNA-10a-5p and *BDNF* in FRDA and CTRL fibroblasts. The regression line between the normalized expression level of the miRNA and BDNF mRNA was calculated with least-squares regression for two sets of measurements. CTRL samples are shown as blue dots, and FRDA samples are shown as red dots

In addition to miRNA regulation of *FXN* expression, changes in the miRNome resulting from decreased levels of *FXN* may affect the expression of numerous other genes. To determine potential miRNA-mediated transcriptome changes, we integrated the results of the miRNAseq and RNAseq analyses [14]. Importantly, both the miRNA and RNAseq analyses were performed on almost identical sets of primary fibroblast lines (29 out of 30 cell lines overlapped), thus allowing direct comparison. We used TargetScan 7.1 and 7.2 to determine the in silico potential targets of the 3 validated differentially expressed miRNAs, miRNA-10a-5p, miRNA-148a-3p, and miRNA-224-5p. A cumulative weighted context++ criterion of − 0.45 or lower was set as a cutoff for putative miRNA-regulated mRNAs. miRNA target transcripts predicted by TargetScan were subsequently compared to the set of genes upregulated or downregulated in the FRDA RNAseq experiment (Venn diagrams in Fig. 5a; Suppl. Fig. 1A; Suppl. Fig. 2A). In the case of miRNA-10a-5p, out of 337 putative targets of this miRNA, 33 were downregulated in the RNAseq experiment while 49 were upregulated (Fig. 5a). For miRNA-148a-3p, the overlap consisted of 55 downregulated and 120 upregulated genes in FRDA (Suppl. Fig. 1A) while for miRNA-224-5p, the respective overlap included 34 and 60 genes (Suppl. Fig. 2A). All statistically significant (*P ≤ 0.05 in unpaired Student’s *t* test) targets are listed in Suppl. Tab. 2 and presented on the heatmaps (Fig. 5b; Suppl. Fig. 1B; Suppl. Fig. 2B).

### *BDNF* Is Targeted by miRNA-10a-5p and Its mRNA Levels Are Downregulated in FRDA

An examination of the genes identified by TargetScan (Suppl. Tab. 2) as putative targets of the miRNAs differentially expressed in FRDA uncovered *BDNF* as a top scoring target of miRNA-10a-5p, the most significantly upregulated miRNA identified in our miRNAseq/qRT-PCR analyses. To confirm whether *BDNF* mRNA expression is indeed decreased in FRDA cells, we validated the RNAseq results by qRT-PCR. The analysis revealed a 2-fold decrease in *BDNF* transcript expression in FRDA patients compared to CTRL subjects (Fig. 5c). Additionally, a negative Spearman’s correlation (rho = 0.51; *P* = 0.006) was observed between the *BDNF* and miRNA-10a-5p expression levels in the CTRL and FRDA cell lines (Fig. 5d). 

**Fig. 6:**
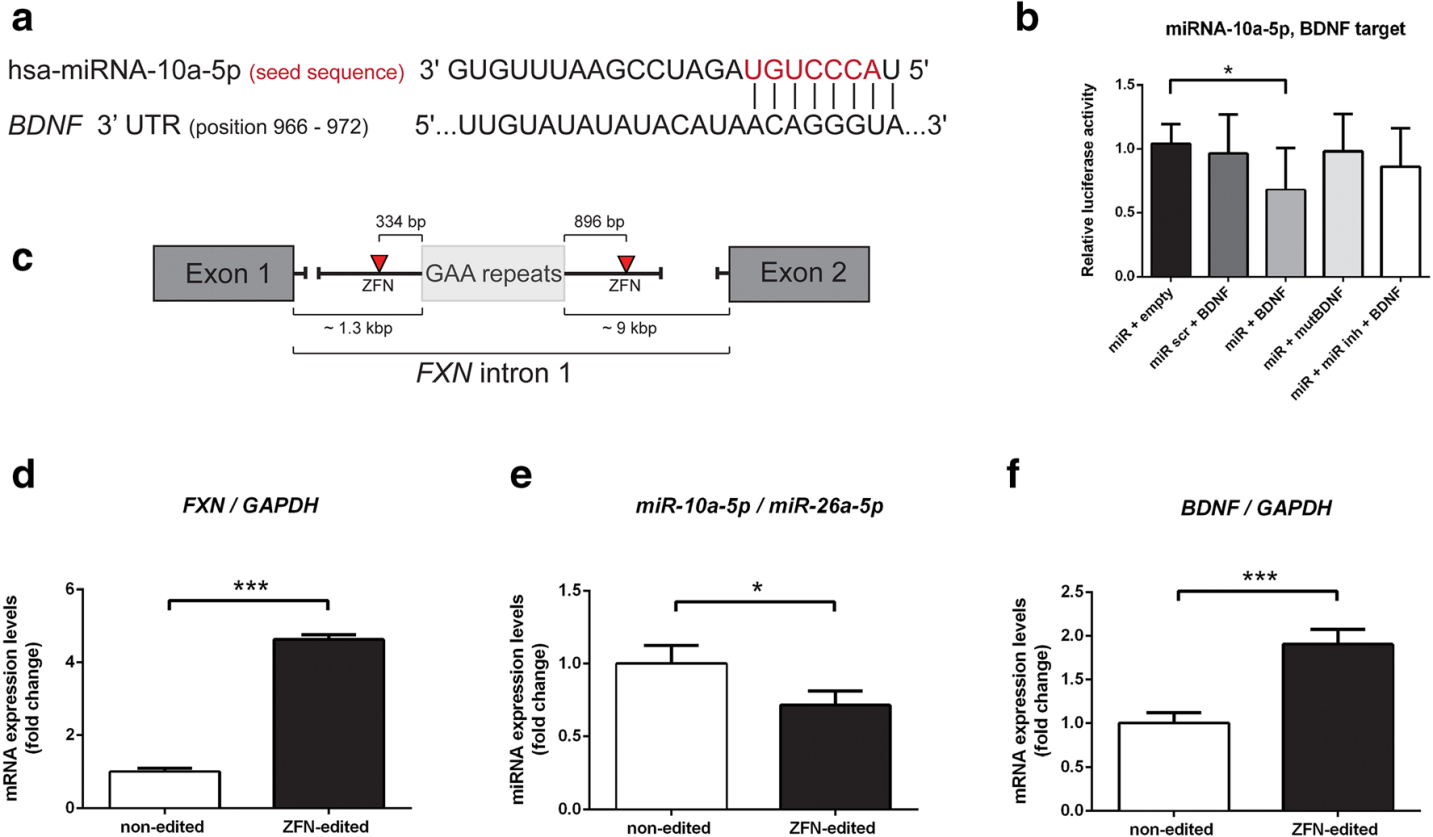
Excision of the expanded GAA repeats decreases miRNA-10a-5p expression and elevates *BDNF* levels. **a** A fragment of the *BDNF* 3′UTR harboring the miRNA-10a-5p target sequence was cloned into a luciferase reporter vector (based on NM_001143805.1, nucleotide positions 966–972). **b** Constructs containing the *BDNF* target sequence and the mutated BDNF target sequence (Suppl. Tab. 1) as well as the empty luciferase reporter vector were co-transfected into HEK293 cells with the appropriate miRNA-10a-5p, unspecific control (miR scr), and miRNA-10a-5p inhibitor as indicated in the graph. Luciferase activity was measured 48 h after transfection. *Firefly* luciferase activity was normalized against *Renilla* luciferase activity. All bars present relative luciferase activity with the standard deviation (SD). Statistical significance was calculated using Student’s *t* test *P ≤ 0.05. **c** Schematic illustrating the strategy of the GAA repeat excision by specific ZFNs (red triangles). Homozygous editing of FRDA fibroblasts was achieved as described in [33]. Homozygous excision of the expanded GAAs increased *FXN* mRNA expression (**d**), decreased miRNA-10a-5p levels (**e**), and consequently upregulated *BDNF* transcript expression (**f**). The results of three independent analyses are shown; *P ≤ 0.05, ***P ≤ 0.001. Comparisons were performed using unpaired Student’s *t* tests. *P ≤ 0.05, ***P ≤ 0.001, n.s. non-significant. Bars present the normalized average fold change compared to CTRL fibroblasts (5 cell lines/group) with standard deviation (SD)

Next, we determined whether miRNA-10a-5p can directly target the *BDNF* 3′UTR at the transcriptional level. In silico analysis of the *BDNF* 3′UTR site revealed one putative binding site for miRNA-10a-5p. A luciferase reporter assay was performed in HEK293 cells, which, according to the miRmine database, express this miRNA at relatively low levels. Empty firefly luciferase vector or vectors cloned with the wild-type or mutant *BDNF* 3′UTR fragments were co-transfected with synthetic hsa-miRNA-10a-5p, hsa-miRNA-10a-5p inhibitor, or control cel-miRNA-239b (miR scr). A schematic of miRNA-10a-5p binding to the *BDNF* 3′UTR and DNA sequences is shown in Fig. 6a and in Suppl. Tab. 1. We found that the luciferase expression levels were diminished upon binding of synthetic miRNA-10a-5p to a luciferase construct with an inserted fragment of the 3′UTR *BDNF* sequence when compared to the vector control (Fig. 6b). Synthetic miRNA-10a-5p had no effect on luciferase reporter expression with the construct containing a seed sequence mutation in the *BDNF* 3′UTR (Fig. 6b; Suppl. Tab. 1).

### Excision of GAA Repeats Increases *FXN* and *BDNF* Expression While Reducing miRNA 10a-5p Abundance

A decreased level of BDNF has been observed in several neurodegenerative diseases [38–40]. To assess whether the expression levels of *FXN*, *BDNF*, and miRNA-10a-5p are interdependent, we edited the expanded GAA repeats from FRDA fibroblasts using ZFNs. Previously, we demonstrated that heterozygous excision of the GAA repeats (removal of one of the expanded GAA tracts) increases *FXN* expression and alleviates some of the phenotypic changes characteristic of FRDA neurons and cardiomyocytes differentiated from induced pluripotent stem cells [33, 41]. To further increase *FXN* expression to a level comparable to that of unaffected cells, we excised a second expanded GAA tract using the same pair of ZFNs as described previously in [33] (Fig. 6c). The corrected cells indeed expressed ~ 5-fold greater levels of *FXN* mRNA than the parental FRDA cells (Fig. 6d). This significant correction of *FXN* levels resulted in downregulation of miRNA-10a-5p (Fig. 6e) and an almost 2-fold increase in *BDNF* mRNA (Fig. 6f). Thus, correction of FRDA cells via genome editing restored not only *FXN* expression but also miRNA-10a-5p and its target *BDNF* to levels observed in the CTRL cohort. These results demonstrated the regulatory interactions of *FXN*/miRNA-10a-5p/*BDNF*, indicating a possible therapeutic opportunity for FRDA.

## Discussion

Dysregulation of miRNA expression contributes to the development and pathology of various neurodegenerative diseases [42–44]. In this work, we compared the results of miRNA profiling with whole transcriptome mRNA sequencing data obtained using the same pool of FRDA patient and CTRL cell lines, thus allowing us to directly correlate miRNome and transcriptome changes. Although hundreds of potential miRNA-mRNA interactions were revealed by these analyses, after validation, we focused on two miRNAs and their targets with potentially high translational relevance for FRDA therapy. First, we showed that miRNA-224-5p, which is elevated in FRDA cells, targets *FXN* and decreases *FXN* mRNA and protein levels. In addition, we found that an increase in the miRNA-10a-5p expression in FRDA fibroblasts is likely responsible for a significant decrease in *BDNF* mRNA levels in patient cells. In silico target predictions, the negative correlation between miRNA and *BDNF* expression and prior studies on *BDNF* regulation [45] strongly support the notion that its level is regulated by miRNA-10a-5p. Moreover, ZFN-mediated excision of the expanded GAA repeats in fibroblasts corrected miRNA-10a-5p overexpression and the *BDNF* deficit.

Uncovering miRNA(s) that efficiently target *FXN* mRNA could lead to the development of potential therapeutic interventions via blocking miRNA-mRNA interactions and consequently upregulating *FXN*. Earlier studies using both in silico and reporter systems demonstrated that the *FXN* 3′UTR can be targeted by miRNA-124-3p [46] and miRNA-886 [27] (later classified as a much larger vault RNA). Although none of these RNAs were dysregulated in FRDA fibroblasts, our TargetScan analyses indicated that 5 out of 13 miRNAs differentially expressed in FRDA fibroblasts have the potential to bind the *FXN* 3′UTR (Figs. 1b and 3a). Validation analyses confirmed that miRNA-224-5p consistently downregulated endogenous *FXN* mRNA and protein levels. Surprisingly, although two putative binding sites for miRNA-224-5p were found within the 3′UTR of the *FXN* mRNA, transfection of miR-224-5p did not affect the luciferase reporter activity, thus indicating that this miRNA exerts its effects on *FXN* indirectly. Importantly, no other potential miRNA-224-5p target sites were identified in the 5′UTR or coding sequence of the *FXN* mRNA using the miRWalk database [47]. We conducted in silico analyses to elucidate the potential connection between miRNA-224-5p and *FXN*. We identified 34 genes that are downregulated in FRDA fibroblasts and represent predicted targets for miRNA-224-5p. Enrichr analyses [48] of this gene set demonstrated a strong overrepresentation of genes involved in RNA polymerase II transcription, gene expression, and chromatin organization categories (Suppl. Fig. 2C). In addition, miRNA-224-5p has been shown to regulate the processes of autophagy and apoptosis [49, 50]. Thus, the indirect effect of miRNA-224-5p on *FXN* expression is likely mediated via targeting of chromatin modifiers, transcription machinery, or by other critical intracellular processes. Increased levels of this miRNA in FRDA cells could also be, at least in part, responsible for the reported global decrease in the transcriptome and proteome in the affected cells [14, 51].

Although the indirect targeting of *FXN* by miRNA-224-5p may constitute an important regulatory pathway affecting *FXN* levels, it does not represent a clear target for potential therapeutic intervention. In contrast, our discovery of miRNA-10a-5p upregulation in FRDA cells may have a direct translational impact through the regulation of *BDNF* levels. Importantly, *BDNF* received the highest probability score among the miRNA-10a-5p targets predicted by TargetScan [35]. BDNF is a neurotrophic factor that regulates the function of the nervous system through various mechanisms, such as the maintenance of neuron development, synaptic plasticity, and neurotransmitter release [52–54]. Expression of the *BDNF* gene is reduced in patients with several neurodegenerative diseases, including Alzheimer’s, Parkinson’s, and Huntington’s disease [38–40]. Importantly, gene transfer of *BDNF* into both primary neurons and a mouse model of FRDA impeded neurodegeneration, underscoring the importance of *BDNF* in this process [55]. In addition, regulation of *BDNF* expression levels by miRNA-10a-5p has been reported previously in granulosa cells of the ovary [56] and cervical cancer cells [45]; this miRNA was also differentially expressed in the cerebrospinal fluid of Alzheimer’s and Parkinson’s disease patients [57] as well as in brain tissue samples from Huntington’s disease patients [58]. Taken together, these data indicate that miRNA-10a-5p is expressed in the nervous system, dysregulated in several neurological conditions, and confirmed to target *BDNF*, which is downregulated in neurodegeneration. On the other hand, Quesada et al. observed an increase in *BDNF* expression in periodontal ligament cells of FRDA patients [28]. However, this is the only study, so far, which shows elevated expression levels of *BDNF* in a FRDA model.

Two main areas of studies dominate translational research efforts of FRDA: discovery and evaluation of new approaches aimed at alleviating FXN deficiency and discovery of disease biomarkers allowing for objective evaluation of disease progression, prognosis, or treatment efficacy [59–61]. miRNAs as small, abundant, and relatively stable molecules, have been evaluated as biomarkers in numerous neurodegenerative disorders, including FRDA [62, 63]. Seco-Servera et al. reported that miRNA-323-3p was highly abundant in the blood of FRDA patients and identified it as a marker for cardiomyopathy [30]. Dantham et al. also identified miRNAs that were differentially represented in FRDA plasma [31]. None of the plasma-specific FRDA miRNAs were found to be differentially expressed in our study, emphasizing tissue specificity as a critical variable in these studies. In addition to being proposed as biomarkers of pathology in neurodegenerative diseases, miRNAs represent attractive therapeutic targets. Current experimental approaches include the use of miRNA mimics (overexpressing miRNA) or antisense oligonucleotides (downregulating miRNA) [64, 65]. Numerous proof-of-concept studies employing miRNAs have been reported for neurological disorders including trinucleotide repeat disorders such as Huntington’s disease and spinocerebellar ataxia 3 [66, 67].

Although this work represents the first direct comparison between miRNA and mRNA transcriptomes obtained from the same set of primary FRDA and CTRL cells, it has certain limitations. The number of samples used herein does not allow for statistical correlations with age or disease severity measures such as FARS. More importantly, despite their primary cell character and the presence of the underlying molecular defect (e.g., GAA expansion and low *FXN* levels), fibroblasts are not an affected cell type in FRDA. Validating our findings in neuronal or cardiac cells would be necessary prior to therapeutic proof-of-concept studies on the miRNA-224-5p/*FXN* and miRNA-10a-5p/*BDNF* interplay. In the case of miRNA-224-5p, further functional work will be necessary to uncover the exact mechanism of its influence on *FXN* levels and assess potential drug targeting of this interaction. It is also important to consider that results obtained using primary cell lines should be carefully evaluated in other model systems and caution should be taken when interpreting their clinical relevance. On the other hand, currently no treatment option exists for FRDA patients, and any novel therapeutic strategy aimed directly or indirectly at correcting FXN downregulation should be evaluated.

## Conclusions

We conducted a comparison of the FRDA transcriptome by integrating the results of mRNA and miRNA sequencing experiments performed using a set of well characterized primary FRDA and CTRL fibroblast lines. We identified and validated differentially expressed miRNAs and, via bioinformatic analyses, identified a putative set of their mRNA targets that are dysregulated in FRDA. We identified miRNA-224-5p, which is upregulated in FRDA cells, as an indirect regulator of *FXN* mRNA and protein levels. In addition, using a luciferase reporter system, we characterized a direct interaction between miRNA-10a-5p (upregulated in FRDA) and *BDNF* (downregulated in FRDA). We demonstrated, using isogenic FRDA and ZFN-corrected fibroblasts, that increased *FXN* expression corrects both miRNA-10a-5p and *BDNF* levels. Combined with results of prior studies on the protective role of BDNF in neuronal degeneration in FRDA models,our study not only validated the miRNA-10a-5p-*FXN*-*BDNF* interplay, but also identified this miRNA as well as *BDNF* as potential therapeutic targets in FRDA.

## Electronic Supplementary Material

Supplementary Figure 1. **Identification of differentially expressed miRNA-148a-3p targets in FRDA cells. (A)** Venn diagram showing an overlap between mRNA differentially expressed in FRDA versus CTRL fibroblasts (yellow – downregulated, blue – upregulated) and TargetScan 7.1-predicted targets of miRNA-148a-3p (red). **(B)** A heatmap illustrates the differential expression of 175 genes (55 downregulated and 120 upregulated, *P ≤ 0.05 in unpaired Student’s t-test) predicted to be targets of the miRNA-148a-3p in FRDA (n=18) versus CTRL (n=17) fibroblasts. The expression level is represented by the colored bars from purple (low expression) to green (high expression). (JPG 2560 kb)

Supplementary Figure 2. **Quantitative RNA profiling of miRNA-224-5p targets. (A)** Venn diagram showing an overlap between mRNA differentially expressed in FRDA versus CTRL fibroblasts (yellow – downregulated, blue – upregulated) and TargetScan 7.2-predicted targets of miRNA-224-5p (red). **(B)** A heatmap illustrates the differential expression of 94 genes (34 downregulated and 60 upregulated, *P ≤ 0.05 in unpaired Student’s t-test) predicted to be targets of miRNA-224-5p in FRDA versus CTRL fibroblasts. The expression level is represented by the colored bars from purple (low expression) to green (high expression). **(C)** Pathways affected by genes downregulated in FRDA and predicted to be targets of miRNA-224-5p. Analyses were conducted using Reactome 2016 in the Enrichr suite [48]. Pathways enriched with *P* ≤ 0.05 are shown. (JPG 3261 kb)

Supplementary Table 1. The sequences of miRNA binding sites cloned into luciferase reporter constructs. Two types of constructs were prepared for miRNA-224-5p and miRNA-10a-5p: wild type and carrying mutations in seed sequence (mut). Two binding sites of miRNA-224-5p were found in 3'UTR region of *FXN,* thus separate constructs were created. Sequences of both sense and antisense strands are written in 5' to 3' direction. Mutated bases are marked in red. (DOCX 13 kb)

Supplementary Table 2. Transcripts downregulated or upregulated in FRDA cells and predicted to be targets of miRNAs: 10a-5p, 148a-3p and 224-5p (FDR<0.05). Downregulated and upregulated transcripts are shown in separate tabs. Columns indicate locus, gene name, average normalized DESeq counts for CTRL, average normalized DESeq counts for FRDA, fold change, p-value and FDR. (XLSX 55 kb)

## Data Availability

The datasets supporting the conclusions of this article are included within the article and its additional files. The RNAseq dataset supporting the conclusions of this article is available at GEO, accession GSE104288.

## Abbreviations

BDNF: Brain-derived neurotrophic factor
CTRL: Control (non-disease carriers)
FRDA: Friedreich’s ataxia
FXN: Frataxin
GAA: Guanine-adenine-adenine
miRNAseq: MicroRNA sequencing
RNAseq: RNA sequencing
qRT-PCR: Quantitative reverse transcriptase real-time PCR
ZFNs: Zinc-finger nucleases
