# Supplementary figures and images for: A Comprehensive Transcriptome Analysis Identifies FXN and BDNF as Novel Targets of miRNAs in Friedreich’s Ataxia Patients
Source: Mol Neurobiol. 2020 Apr 14;57(6):2639–53. doi: 10.1007/s12035-020-01899-1 (PMC7253519; doi:10.1007/s12035-020-01899-1)

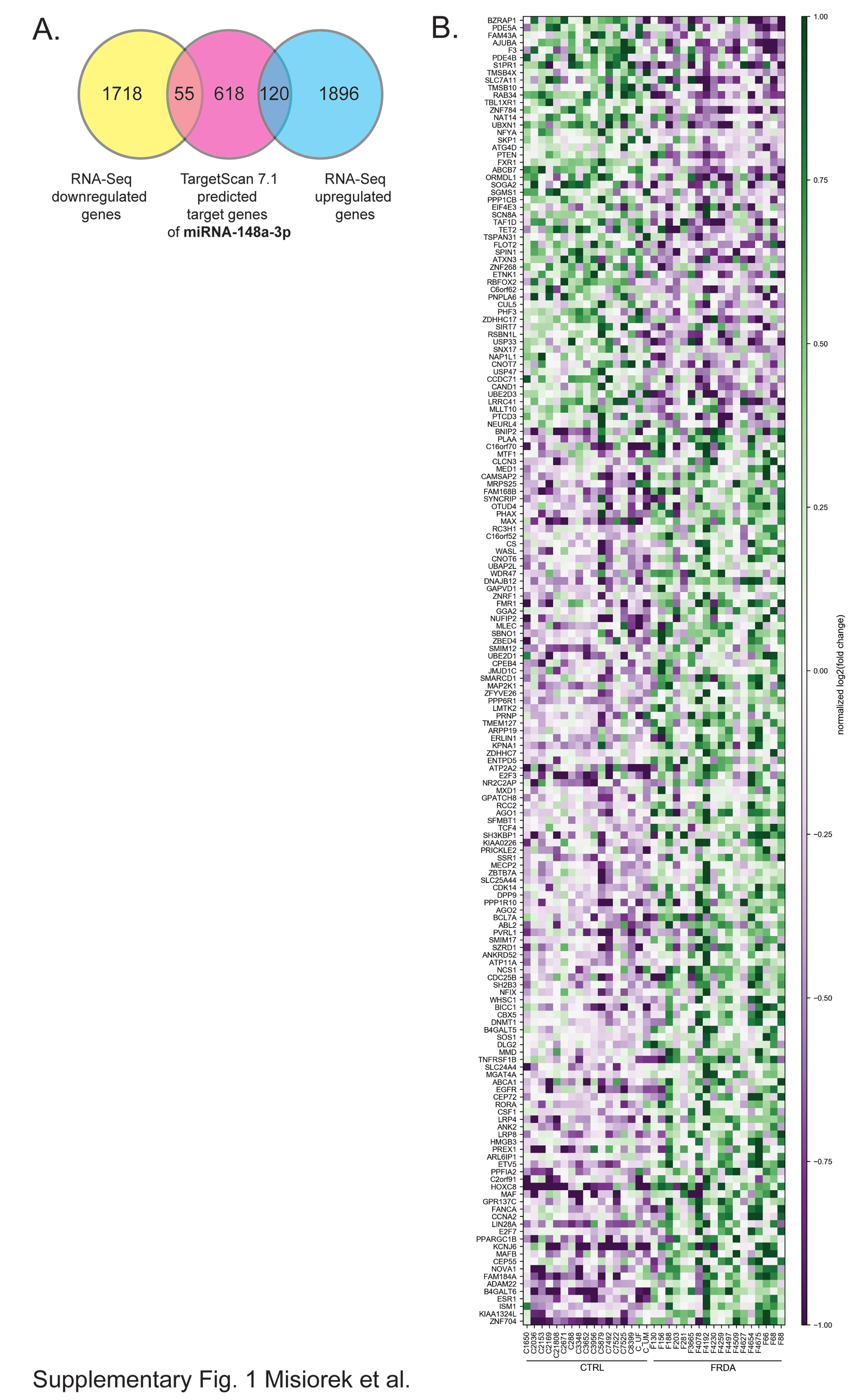

Supplement: Supplementary file 1 — Identification of differentially expressed miRNA-148a-3p targets in FRDA cells. (A) Venn diagram showing an overlap between mRNA differentially expressed in FRDA versus CTRL fibroblasts (yellow – downregulated, blue – upregulated) and TargetScan 7.1-predicted targets of miRNA-148a-3p (red). (B) A heatmap illustrates the differential expression of 175 genes (55 downregulated and 120 upregulated, *P ≤ 0.05 in unpaired Student’s t-test) predicted to be targets of the miRNA-148a-3p in FRDA (n=18) versus CTRL (n=17) fibroblasts. The expression level is represented by the colored bars from purple (low expression) to green (high expression). (JPG 2560 kb) [file 12035_2020_1899_MOESM1_ESM.jpg]

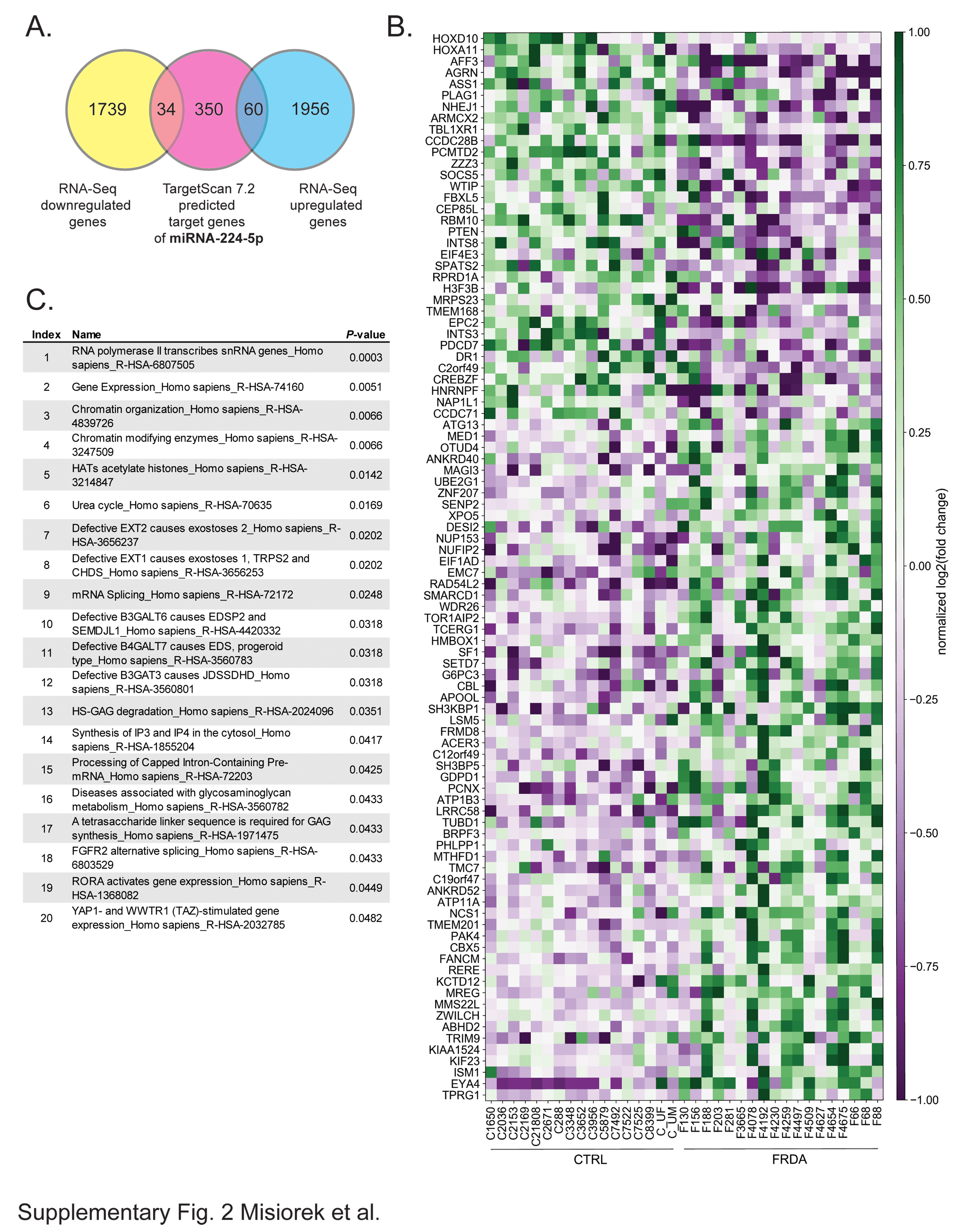

Supplement: Supplementary file 2 — Quantitative RNA profiling of miRNA-224-5p targets. (A) Venn diagram showing an overlap between mRNA differentially expressed in FRDA versus CTRL fibroblasts (yellow – downregulated, blue – upregulated) and TargetScan 7.2-predicted targets of miRNA-224-5p (red). (B) A heatmap illustrates the differential expression of 94 genes (34 downregulated and 60 upregulated, *P ≤ 0.05 in unpaired Student’s t-test) predicted to be targets of miRNA-224-5p in FRDA versus CTRL fibroblasts. The expression level is represented by the colored bars from purple (low expression) to green (high expression). (C) Pathways affected by genes downregulated in FRDA and predicted to be targets of miRNA-224-5p. Analyses were conducted using Reactome 2016 in the Enrichr suite [48]. Pathways enriched with P ≤ 0.05 are shown. (JPG 3261 kb) [file 12035_2020_1899_MOESM2_ESM.jpg]
