# Supplementary material for: A Comprehensive Transcriptome Analysis Identifies FXN and BDNF as Novel Targets of miRNAs in Friedreich’s Ataxia Patients
Source: Mol Neurobiol. 2020 Apr 14;57(6):2639–53. doi: 10.1007/s12035-020-01899-1 (PMC7253519; doi:10.1007/s12035-020-01899-1)
Supplement: Supplementary file 3 — The sequences of miRNA binding sites cloned into luciferase reporter constructs. Two types of constructs were prepared for miRNA-224-5p and miRNA-10a-5p: wild type and carrying mutations in seed sequence (mut). Two binding sites of miRNA-224-5p were found in 3'UTR region of FXN, thus separate constructs were created. Sequences of both sense and antisense strands are written in 5' to 3' direction. Mutated bases are marked in red. (DOCX 13 kb) [file 12035_2020_1899_MOESM3_ESM.docx]

**Supplementary Table 1. The sequences of miRNA binding sites cloned into luciferase reporter constructs.**

| **miRNA** | **Target** | **Construct name** | **Strand** | **Inserts: Sequence 5’ – 3’ with seed sequence  and mutated bases** |
| --- | --- | --- | --- | --- |
| **224-5p** | ***FXN*** | **FXN1** | Sense | AAATAGCGGCCGCTAGTATTTGTGCTCTGTGACTGCCT |
|  |  |  | Antisense | CTAGAGGCAGTCACAGAGCACAAATACTAGCGGCCGCTATTT |
|  |  | **FXN2** | Sense | AAATAGCGGCCGCTAGTCTGTAACCTGGGTGACTGAGT |
|  |  |  | Antisense | CTAGACTCAGTCACCCAGGTTACAGACTAGCGGCCGCTATTT |
|  |  | **mutFXN1** | Sense | AAA TAGCGGCCGCTAGTATTTGTGCTCTATAAGGGCCT |
|  |  |  | Antisense | CTAGAGGCCCTTATAGAGCACAAATACTAGCGGCCGCTATTT |
|  |  | **mutFXN2** | Sense | AAATAGCGGCCGCTAGTCTGTAACCTGGCTAATGGAGT |
|  |  |  | Antisense | CTAGACTCCATTAGCCAGGTTACAGACTAGCGGCCGCTATTT |
| **10a-5p** | ***BDNF*** | **BDNF** | Sense | AAATAGCGGCCGCTAGTTATATATACATAACAGGGTAAAT |
|  |  |  | Antisense | CTAGATTTACCCTGTTATGTATATATAACTAGCGGCCGCTATTT |
|  |  | **mutBDNF** | Sense | AAATAGCGGCCGCTAGTTATATATACATATCGGCGCAAAT |
|  |  |  | Antisense | CTAGATTTGCGCCGATATGTATATATAACTAGCGGCCGCTATTT |
